# Omics research in the field of heart failure: A bibliometric analysis from 2008 to 2024

**DOI:** 10.1097/MD.0000000000045234

**Published:** 2025-10-24

**Authors:** Lin Zhou, Zihan Wang, Jiankang Wang, Hang Shang, Wenxuan Wang, Jiayu Mao, Yongsheng Huang, Yingzi Cui, Jiajuan Guo

**Affiliations:** a College of Traditional Chinese Medicine, Changchun University of Chinese Medicine, Changchun, China; b Department of Cardiology, The Affiliated Hospital to Changchun University of Chinese Medicine, Changchun, China.

**Keywords:** bibliometrics, bioinformatics, biomakers, heart failure, omics

## Abstract

**Objective:**

To conduct a bibliometric analysis of heart failure (HF) omics research from 2008 to 2024 using CiteSpace, exploring research hotspots and the latest advancements in this field.

**Methods:**

Literature on HF omics research published between January 2008 and December 2024 was retrieved from the web of science core collection. Microsoft Excel 2019 was used to calculate publication volume and growth trends, while CiteSpace 6.2.R4 was employed to generate visual maps and conduct visual analyses of authors, institutions, countries, journals, references, and keywords.

**Results:**

A total of 282 articles were included for bibliometric analysis. The results indicate a gradual increase in the number of publications on HF omics research over the past 16 years. The leading research countries in this field are the United States, China, the United Kingdom, Germany, and Italy. Authoritative research institutions include the German Center for Cardiovascular Research, Harvard University, and Brigham and Women’s Hospital. The representative researcher is Zannad, Faiez. Key research keywords include biomarkers, coronary heart disease (CHD), pathways, receptors, acute myocardial infarction (AMI), and atrial fibrillation (AF).

**Conclusion:**

Omics Research in the Field of HF has been steadily increasing year by year. In terms of research directions, hotspots often involve studies related to CHD, AMI, AF, and other associated conditions. Regarding research objectives, the primary focuses include identifying biomarkers, achieving precision medicine, and elucidating gene expression mechanisms.

## 1. Introduction

Heart failure (HF) is a severe manifestation or end-stage condition of various cardiac diseases. It is a complex clinical syndrome characterized primarily by symptoms such as dyspnea, fatigue, and fluid retention, resulting from impaired ventricular filling or ejection function.^[1]^ HF is a global epidemic, affecting approximately 1 to 3% of the world’s population, with around 64.3 million people suffering from it.^[2]^ It is one of the leading causes of hospitalization for patients aged 65 and older, accounting for about 1 to 2% of total hospital admissions in the Western world.^[3]^ left ventricular ejection fraction (EF) is a critical factor in the classification of HF patients, and most clinical trials use EF to select participants. Recent guidelines have introduced the concept of HF with mildly reduced EF, in addition to distinguishing between HF with reduced EF and HF with preserved EF.^[1]^

The treatment landscape for HF is rapidly evolving. The cornerstone medications have expanded from the traditional “golden triangle” to a new paradigm referred to as the “five golden flowers,” which include renin-angiotensin system inhibitors, beta-blockers, mineralocorticoid receptor antagonists, sodium-dependent glucose transporters 2 inhibitors, and soluble guanylate cyclase stimulators.^[4]^ In summary, HF remains a focal point in global cardiovascular research, with continuous advancements in diagnostic and therapeutic approaches. However, its pathogenesis and progression mechanisms are complex and not yet fully understood. Beyond ischemic causes, factors such as valvular heart disease, hypertension, arrhythmias, and substance abuse also contribute to HF.^[5,6]^ With growing insights into cardiac injury patterns (including infection and inflammation), cardiotoxicity, and myocardial metabolic abnormalities, research into the mechanisms underlying HF is becoming increasingly in-depth.

Omics technologies enable the study of as many biomolecules as possible within a sample, offering significant potential for uncovering new biological insights. By examining multiple dimensions such as genes, proteins, metabolites, and metabolic rates, omics approaches address the limitations of traditional research methods, which often focus on a few molecules and fail to systematically explain biological phenomena. Currently, omics technologies are widely applied to explore the pathogenesis of different types of HF, identify diagnostic biomarkers, and investigate the mechanisms of interventions.^[7]^ As such, omics has become a focal point in contemporary basic research on HF.

With the continuous advancement of HF omics research, there is still a lack of comprehensive analysis in this field. Bibliometrics, which employs quantitative statistical methods to analyze published literature, can objectively and comprehensively present emerging frontiers compared to traditional literature reviews, thereby providing guidance for future research.^[8]^ Based on this, this study aims to apply bibliometric and visual analysis methods to synthesize research frontiers in this field, uncovering the temporal and spatial distribution of such research and identifying key contributors. The study seeks to address the following questions: Identify the countries, institutions, journals, and authors that play significant roles in global HF omics research; Reveal the current hotspots and latest advancements in HF omics research.

## 2. Methods

### 2.1. Data source

The web of science core collection (WoSCC) covers a wide range of disciplines, offering extensive literature with high citation rates. Additionally, the CiteSpace software is highly compatible with the literature format exported from the WoSCC database. Therefore, WoSCC was selected as the source of literature for this study.

### 2.2. Data filtering

Inclusion Criteria: Published or accepted in the WoSCC; Research subjects focused on HF; Research methods involving omics studies.

Exclusion Criteria: Low relevance to HF omics research; Popular science articles, call for papers, etc; Duplicate publications; Incomplete information such as year, author, institution, or keywords.

### 2.3. Data fetrieval

**Figure 1.**
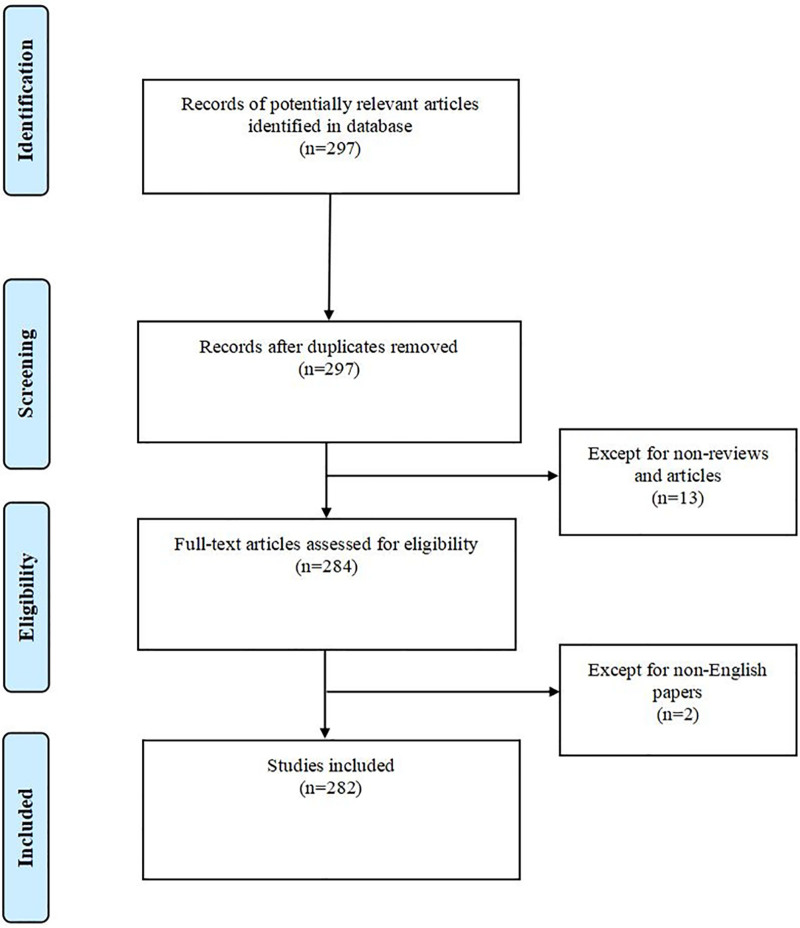
Flow Chart of literature screening. The PRISMA-compliant literature selection flowchart systematically outlines the identification, screening, eligibility assessment, and inclusion procedures of heart failure omics-related publications retrieved from the Web of Science (WOS) core collection.

The search query was formulated as: “TS = (HF) AND TS = (omics).” The retrieval period spanned from January 1, 2008, to December 31, 2024, with document types limited to “Article” and “Review” and language restricted to English. After deduplication, a total of 282 articles were included for analysis. The flowchart of this study is illustrated in Figure 1.

### 2.4. Data transformation

Microsoft Excel 2019 was utilized to calculate the annual publication output and growth trends. CiteSpace 6.2.R4 software was employed to generate network maps, creating visual network diagrams for authors, institutions, countries, journals, references, and keywords, thereby enabling network visualization analysis.

### 2.5. Parameter settings

The parameter settings for CiteSpace are as follows: the time span is set from January 2008 to December 2024, with a selection interval of 1 year; text processing includes term sources (title, abstract, author, keywords) and term types; node types include author, institution, country, cited journal, and keywords.

### 2.6. Ethics section

This study employs bibliometric methods for data analysis. All data utilized were publicly available on the internet and did not involve animals, humans, or any biological samples, thus obviating the need for approval from an ethics committee.

## 3. Results

### 3.1. Statistics on the number of papers published

**Figure 2.**
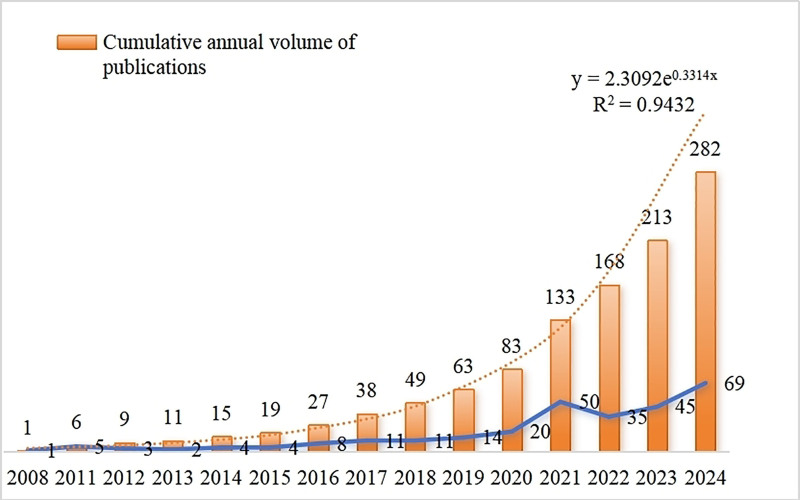
Number of papers published per year. The scientometric chart displays the chronological distribution of publications over the 2008–2024 timeframe, facilitating temporal analysis of scholarly activity trends in this research area.

After a rigorous selection process, 282 valid articles were obtained for data statistics and trend analysis (Fig. 2). The first article included was published in 2008, with an average annual publication volume of 17.6 articles over the past 16 years. The number of publications showed a steady increase from 2008 to 2018, followed by a rapid growth from 2019 to 2021. Although there was a slight decline in 2022 and 2023, the number of publications peaked at 69 in 2024. The cumulative number of articles published each year was exponentially fitted using a growth trend model with *R*^2^ = 0.9498. The fitting curve equation is *y* = 2.3092e^0.3314*x*^, indicating a steady upward trend in the number of articles published in the field of HF omics research over the past 16 years.

### 3.2. Country & institutional analysis

**Figure 3.**
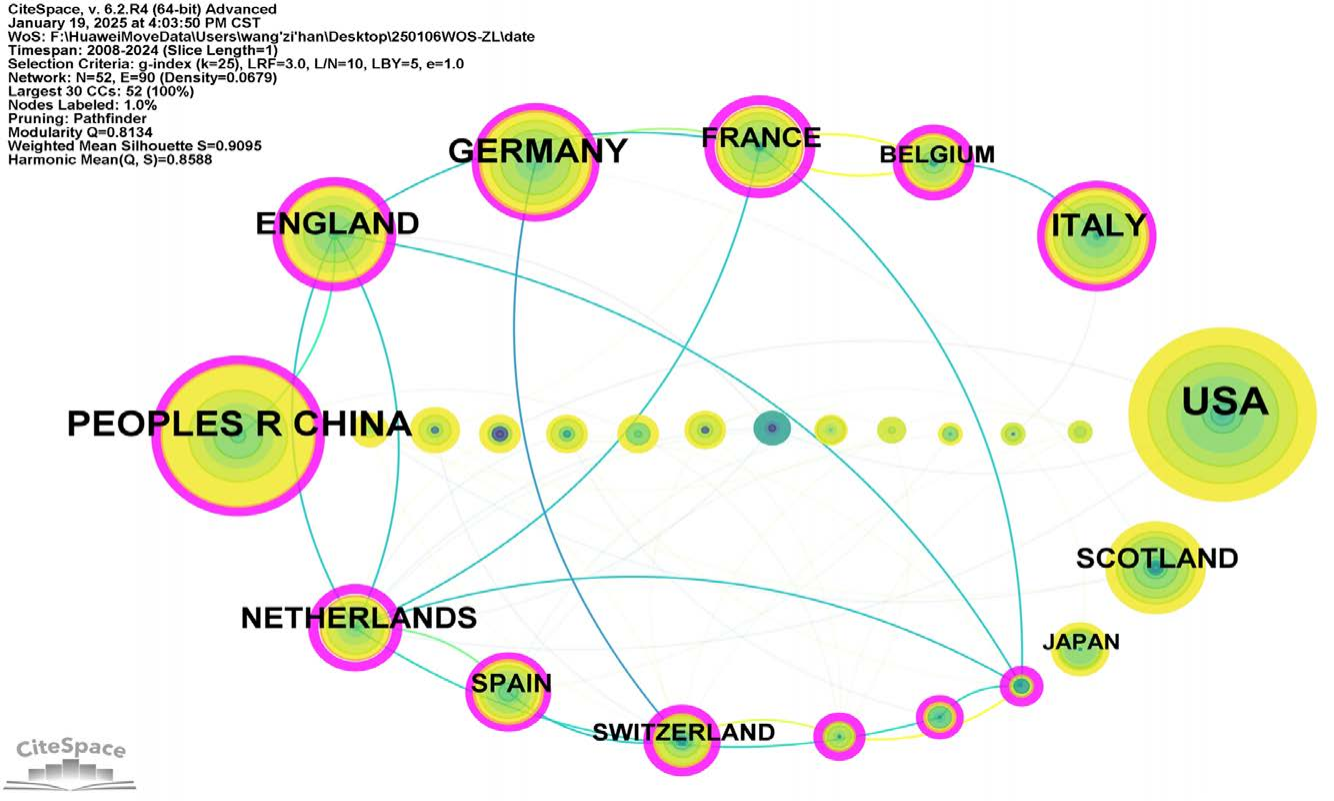
Network diagram of participating countries. The publication volume of each country is represented by the size of its corresponding circle. The connecting lines between circles indicate international collaborations among countries, where thicker and more numerous lines denote stronger collaborative relationships. The pink outer ring represents the betweenness centrality, with a thicker pink ring indicating higher betweenness centrality.

**Figure 4.**
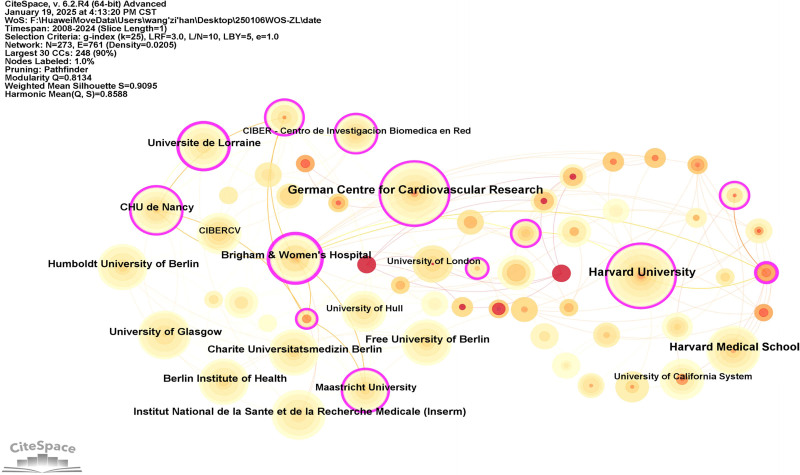
Network diagram of participating publishers. The visualization principle aligns with the country collaboration network. The publication output of research institutions is represented by circle size, while inter-institutional collaboration is indicated by connecting lines. Thicker and more frequent lines denote stronger collaborative relationships between institutions. The pink outer ring denotes betweenness centrality, with a thicker ring reflecting higher betweenness centrality.

A occurrence analysis was conducted on the countries and institutions involved in the publication of research literature, resulting in the formation of collaborative network diagrams (Figs. 3 and 4). A total of 52 countries and 273 institutions were included. There were 32 countries with a publication volume of ≥3 articles. The top 5 countries by publication volume were the United States (108 articles, 38.3%), China (68 articles, 24.1%), Germany (43 articles, 15.2%), Italy (38 articles, 13.5%), and the United Kingdom (36 articles, 12.8%). Among these 5 countries, the United States was the earliest to publish and had the highest number of publications, while China was the only Asian country, demonstrating the significant influence of countries in Europe and America in this field. Additionally, the top 5 countries by centrality were the United Kingdom, China, the United States, Italy, and Spain. The United States, China, and the United Kingdom ranked in the top 5 for both publication volume and centrality, indicating that these 3 countries are leading in the field of HF omics research. The top 8 institutions by publication volume are listed in Table 1, with the top 3 being the German Centre for Cardiovascular Research (25 articles), Harvard University (25 articles), and Harvard Medical School (19 articles). Among all institutions, Brigham & Women’s Hospital (16 articles) had the highest centrality (0.30). These 4 institutions are authoritative in the field of HF omics research.

**Table 1:** Top 8 institutions in terms of publications.

Ranking	Institution	Count	Centrality	Country
1	Harvard University	25	0.09	Armenia
2	German Centre for Cardiovascular Research	25	0.06	Germany
3	Harvard Medical School	19	0.01	Armenia
4	Institut National de la Sante et de la Recherche Medicale (Inserm)	18	0.10	France
5	University of Glasgow	17	0.02	England
6	Brigham & Women’s Hospital	16	0.30	Armenia
7	CHU de Nancy	16	0.11	France
8	Berlin Institute of Health	15	0.03	Germany

### 3.3. Authors collaborated on the analysis

**Figure 5.**
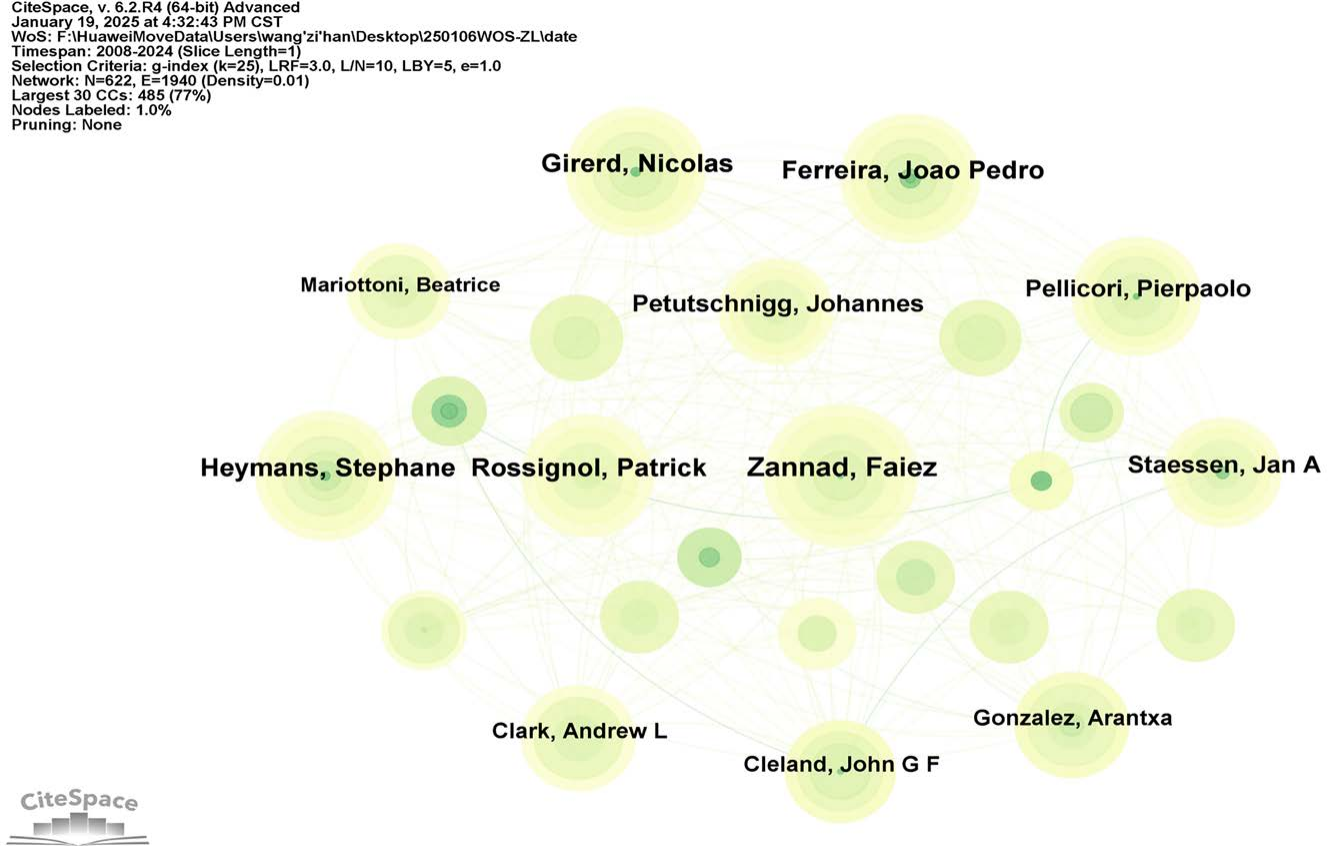
Network diagram of the authors who participated in the posting. In this bibliometric analysis of heart failure omics research, we constructed a co-authorship network where node size corresponds to author productivity (quantified by publication count), edges represent collaborative relationships between researchers, and edge weight (reflected by line thickness) indicates collaboration intensity – with thicker lines denoting stronger coauthor ties, thereby enabling identification of key opinion leaders (large nodes), research consortia (densely connected clusters), and interdisciplinary bridges (high-betweenness nodes).

A total of 622 contributing authors were included, with the top ten authors by publication volume listed in Table 2. The most prolific author is Zannad, Faiez (18 articles), followed by Rossignol, Patrick (14 articles). Using Citespace, an author collaboration network diagram was generated (Fig. 5), with the node threshold set to 0.1. It was found that the centrality of collaborations among authors was <0.1, indicating a relatively weak level of collaboration among authors in this field. Future research may benefit from enhanced collaborative efforts. Omics research, serving as a bridge between basic and applied research, has emerged as a cutting-edge technology with extensive applications. Beyond the medical field, scholars in disciplines such as chemistry, pharmaceutical sciences, computer science, and artificial intelligence have also begun employing omics technologies for related research. Consequently, researchers have been unable to establish efficient collaboration and communication in the short term. Currently, big data-driven interdisciplinary collaboration has gradually become a dominant research paradigm. We attempted to analyze this collaborative model but failed to derive significant findings. Nevertheless, given the unique translational value of omics research, we anticipate that, over time, scholars in the field of HF omics research will inevitably develop tight-knit academic exchanges.

**Table 2:** Top 10 authors in terms of publications.

Ranking	Author	Year	Count	Centrality
1	Zannad, Faiez	2016	18	0
2	Rossignol, Patrick	2016	14	0
3	Ferreira, Joao Pedro	2016	13	0
4	Girerd, Nicolas	2016	13	0
5	Heymens, Stephane	2016	13	0.01
6	Pellicori, Pierpaolo	2016	12	0
7	Staessen, Jan A	2016	11	0
8	Petutschnigg, Johannes	2021	10	0
9	Cleland, John G F	2017	9	0.01
10	Gonzalez, Arantxa	2020	9	0.03

### 3.4. Journal analysis

**Figure 6.**
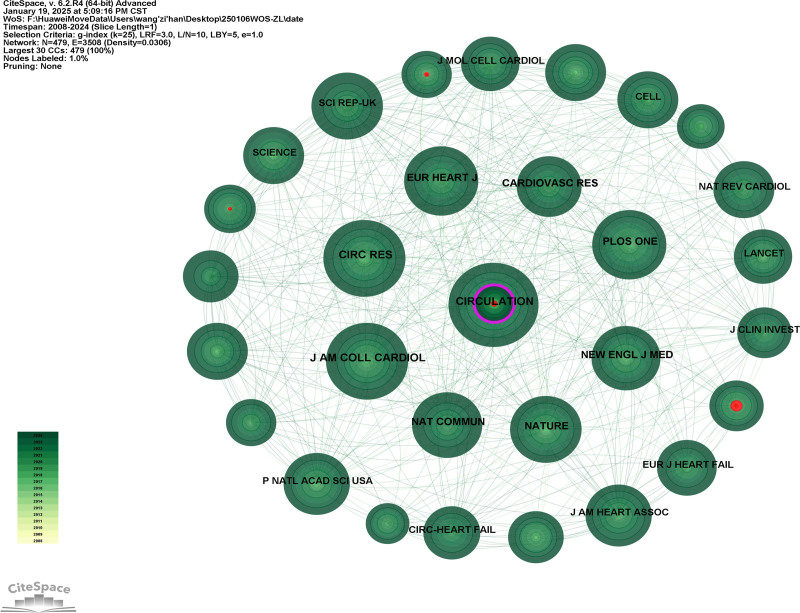
Cited journal collaboration network diagram. We constructed a journal co-citation network for bibliometric analysis, where node size represents citation frequency (larger circles indicating higher citation counts), connecting edges denote inter-journal collaborative relationships (with thicker lines reflecting stronger cooperation intensity), and pink concentric rings visualize betweenness centrality (thicker rings indicating greater intermediary influence in the knowledge network), thereby enabling identification of core journals (highly cited nodes), interdisciplinary bridges (high-betweenness nodes), and collaborative clusters (densely connected journal groups) within the academic landscape.

A total of 169 journals have published articles on HF omics research. The top eleven journals by the number of publications in this field are listed in Table 3. The average impact factor of these 11 journals is 13.37, indicating a high quality of research output in this field. Among them, the journal Frontiers in Cardiovascular Medicine has the highest number of publications (10 articles), and several high-quality journals are included. Among the journals with ≥3 publications, the journal with the highest impact factor is European Heart Journal (38.1). In terms of journal co-citation, a co-occurrence analysis was conducted on all co-cited journals, resulting in a collaborative network diagram (Fig. 6). The top ten journals by co-citation frequency are listed in Table 4. Circulation has the highest number of citations (228 times), followed by Circulation Research (183 times) and Journal of the American College of Cardiology (182 times). The centrality of collaboration among these journals is generally suboptimal.

**Table 3:** Top 11 frequency journals in terms of publications.

Ranking	Journal	Frequency	IF (2024)	*Q* (2024)
1	Frontiers in Cardiovascular Medicine	10	2.8	Q2
2	Scientific Reports	7	3.8	Q1
3	Circulation Research	7	16.5	Q1
4	International Journal of Molecular Sciences	7	4.9	Q1
5	European Journal of Heart Failure	7	16.9	Q1
6	Journal of Translational Medicine	6	6.1	Q1
7	Cardiovascular Research	6	10.4	Q1
8	Heart Failure Reviews	6	4.5	Q1
9	European Heart Journal	6	38.1	Q1
10	Circulation	5	35.6	Q1
11	Basic Research in Cardiology	5	7.5	Q1

**Table 4:** Top 10 frequency cited journals in terms of publications.

Ranking	Cited Journals	Frequency	Centrality	IF (2024)	*Q* (2024)
1	Circulation	228	0.01	35.6	Q1
2	Circulation Research	183	0.07	16.5	Q1
3	Journal of the American College of Cardiology	182	0	21.7	Q1
4	European Heart Journal	157	0.01	38.1	Q1
5	PLOS One	121	0	2.9	Q1
6	Nature	144	0.02	50.5	Q1
7	New England Journal of Medicine	136	0	96.3	Q1
8	Cardiovascular Research	126	0.01	10.4	Q1
9	Nature Communications	125	0.01	14.7	Q1
10	Scientific Reports	125	0.02	3.8	Q1

### 3.5. Keyword analysis

#### 3.5.1. Keyword occurrence

**Figure 7.**
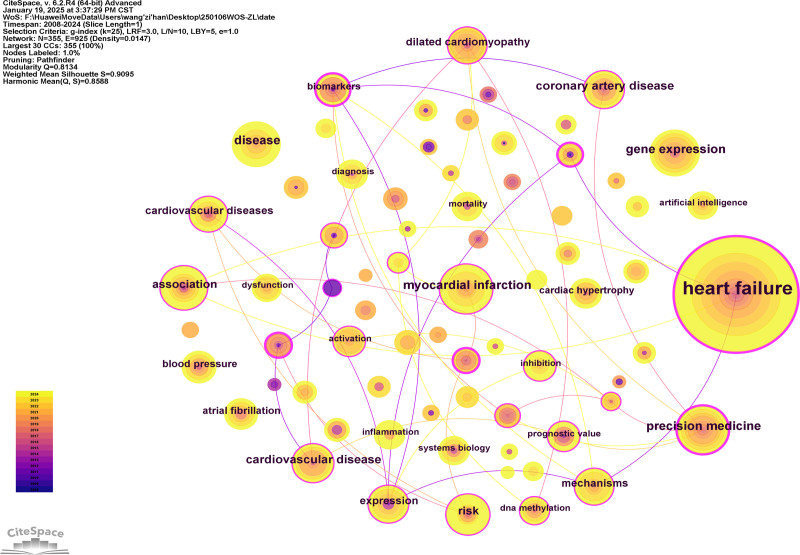
Keyword co-occurrence diagram. This burst detection analysis visualizes high-frequency keywords from relevant literature, where distinct nodes represent individual keywords with node diameter proportional to centrality metrics (larger circles indicating greater conceptual importance), connecting edges demonstrate keyword co-occurrence relationships (thicker and more numerous lines reflecting stronger semantic associations), and pink concentric rings denote betweenness centrality (with annular thickness corresponding to a term’s brokerage potential in the knowledge network), thereby enabling identification of: core concepts (high-centrality nodes), conceptual clusters (densely connected groups), and interdisciplinary bridge terms (high-betweenness keywords) within the heart failure omics research domain.

A statistical analysis of keywords was conducted on the 282 included articles to reveal the core hotspots in this research direction. A total of 355 keywords were identified and used to generate a network co-occurrence diagram (Fig. 7). Keywords with a frequency of ≥10 and a centrality of ≥0.1 were filtered and ranked, as listed in Table 5, representing the hotspots and key aspects of HF omics research. The top 3 keywords are HF (centrality 0.27, frequency 157), precision medicine (centrality 0.17, frequency 26), and gene expression (centrality 0.15, frequency 25). These keywords confirm that HF omics research often uses gene expression as an entry point to explore the mechanisms of HF development and progression, with the aim of achieving precision medicine.

**Table 5:** Top 10 Most frequent keywords in publications (centrality > 0.1).

Ranking	Keywords	Frequency	Centrality
1	Heart failure	157	0.27
2	Precision medicine	26	0.17
3	Gene expression	25	0.15
4	Coronary artery disease	22	0.11
5	Cardiovascular disease	21	0.14
6	Dilated cardiomyopathy	16	0.14
7	Expression	16	0.1
8	Atrial fibrillation	13	0.11
9	Biomarkers	12	0.15
10	Cardiac hypertrophy	11	0.1

#### 3.5.2. Keyword clustering

**Figure 8.**
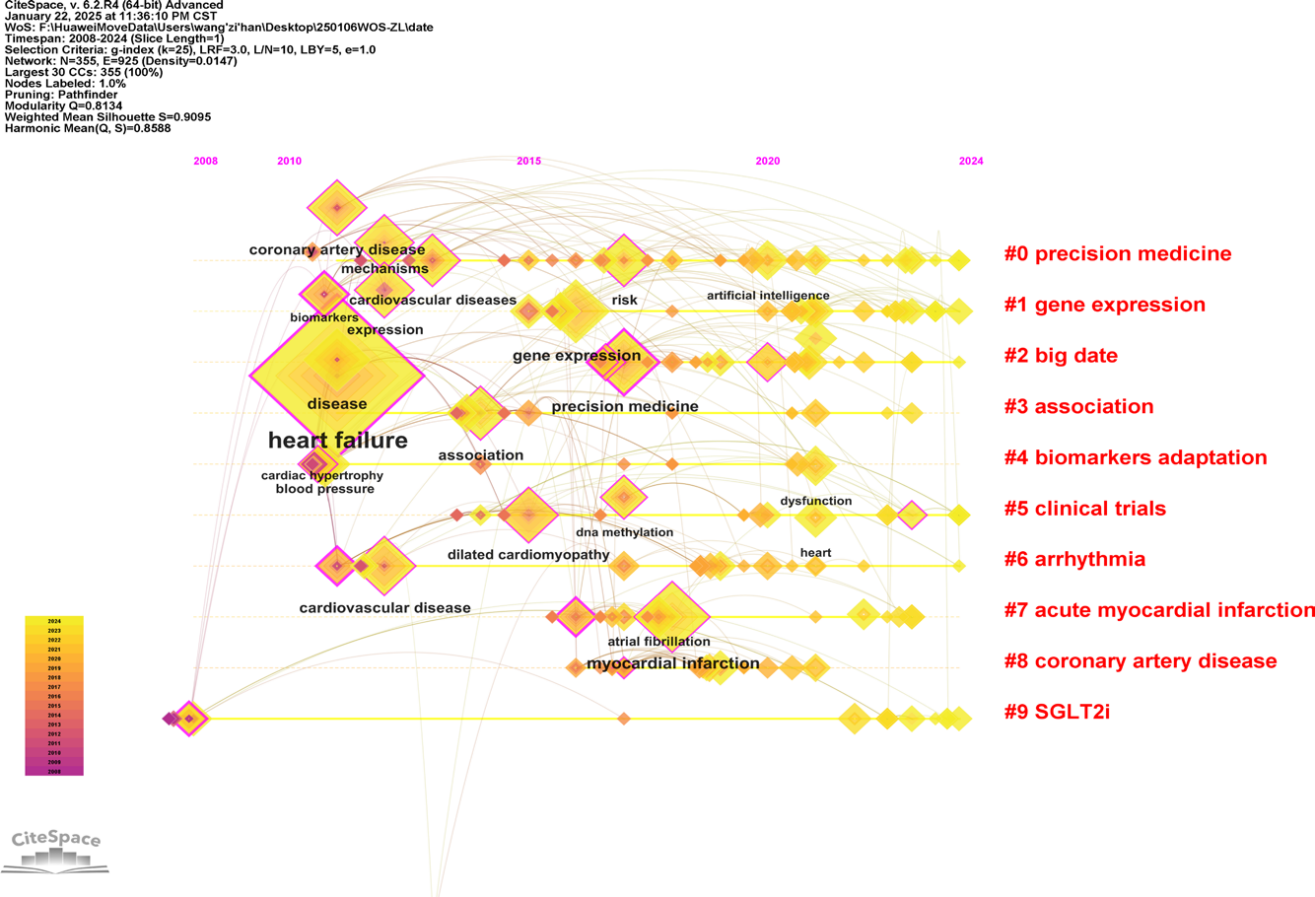
Timeline view of the keyword cluster analysis of the original papers. This timeline visualization presents a chronological keyword co-occurrence analysis, featuring: a temporal axis (top of graph) marking publication years; citation nodes distributed left-to-right along the axis according to initial publication dates (earliest to most recent); diamond-shaped markers representing distinct keywords, where larger diamond size and thicker pink concentric rings (denoting betweenness centrality) indicate greater terminological significance; and cluster labels prefixed with “#” to identify thematic groupings, collectively enabling temporal tracking of conceptual evolution and emerging research fronts in the target domain.

Building upon the keyword co-occurrence analysis of HF omics research literature, the LLR algorithm was employed to conduct a cluster analysis of keywords. The size of the clustering modules is inversely proportional to the cluster sequence number, with the largest clustering module labeled as #0, and the sizes of other clusters following suit, resulting in Timeline view of the keyword cluster analysis (Fig. 8). The modularity value (Modularity Q, Q value) of the cluster analysis network is 0.8134, and the mean silhouette value (Mean Silhouette, S value) is 0.9095. According to the clustering effectiveness indicators, the cluster analysis results of this study are demonstrated to be of high quality, efficient, and reliable. The representative terms of the top ten largest clusters are precision medicine, gene expression, big data, association, biomarkers adaptation, clinical trials, acute myocardial infarction (AMI), coronary artery disease, and sodium-dependent glucose transporters 2 inhibitors, indicating that these ten terms hold significant importance in HF omics research.

#### 3.5.3. Keyword with the strongest citation bursts

**Figure 9.**
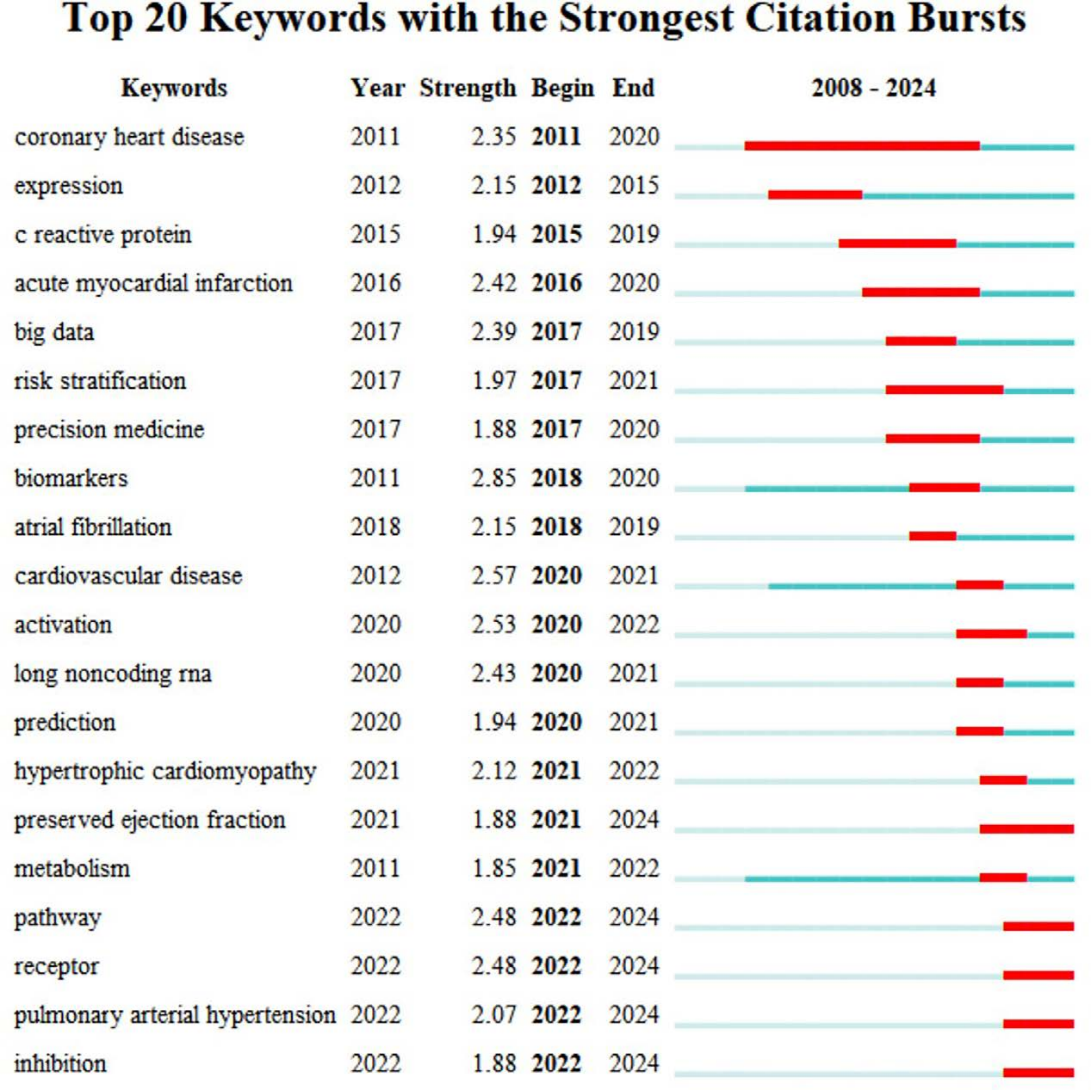
Top 20 keywords with the strongest citation bursts. The keyword citation burst analysis serves as an effective methodology for detecting rapid surges in academic interest toward specific research topics. In the visualization, red lines demarcate the duration of keyword bursts, while blue lines indicate intervals between these bursts. By identifying the top 20 most frequently cited keywords, this analysis reveals which thematic areas garnered concentrated scholarly attention during particular time periods. The occurrence of such bursts signifies that corresponding topics have emerged as academic foci within short temporal spans, reflecting both heightened intellectual engagement and vigorous research activity within the scholarly community. This analytical approach effectively captures paradigm shifts and evolving research priorities in the target domain.

Based on the keyword clustering analysis results, the top 20 high-frequency keywords from recent years were used to generate burst terms (Fig. 9), thereby identifying the frontier hotspots and future trends in research. Among these, “coronary heart disease (CHD),” “AMI,” and “atrial fibrillation (AF)” are burst terms with early high intensity and long duration, indicating that a history of conditions such as CHD, AF, and AMI serves as an important research background or baseline data in HF omics studies. In recent years, keywords such as “biomarker” (2.85), “pathway” (2.48), and “receptor” (2.48) have shown high intensity, suggesting that current HF omics research has progressively delved into exploring disease mechanisms. The study of biomarkers and the precise mechanisms of HF has become a mainstream focus.

### 3.6. Co-cited literature analysis

**Figure 10.**
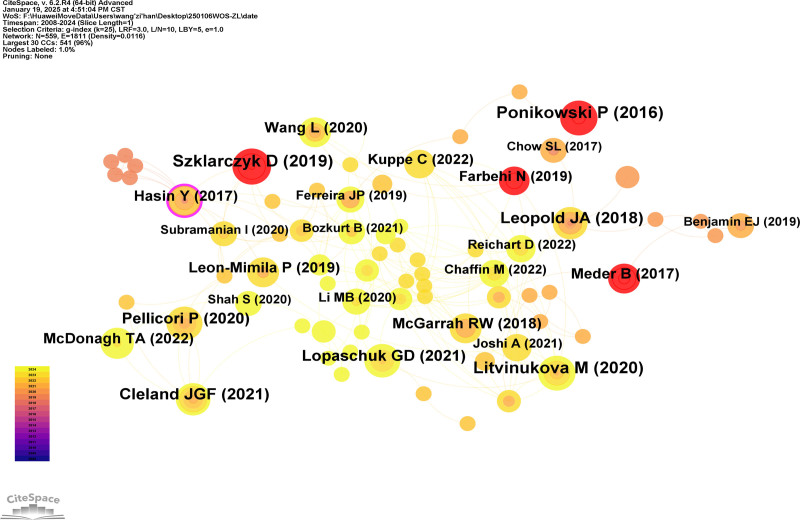
Co-cited reference diagram. The citation network visualization employs a chromatic gradient (yellow–orange–red) to represent citation frequency, with color saturation corresponding to citation intensity – pale yellow indicating lower citation counts, transitioning through orange to deep red signifying higher citation frequencies, thereby enabling immediate visual identification of seminal works (red nodes) versus less cited references (yellow nodes) within the academic landscape.

**Figure 11.**
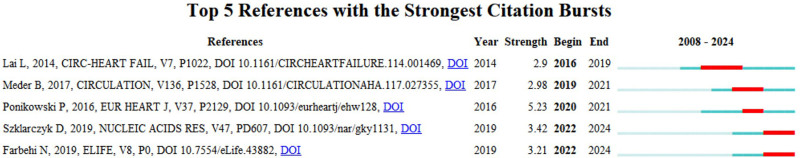
Top 5 references with the strongest citation bursts. The keyword citation burst analysis serves as a robust bibliometric method for identifying authoritative references in this field, where red lines demarcate the duration of keyword bursts and blue lines indicate inter-burst intervals. The top 5 most-cited publications represent the focal literature that has garnered predominant attention from researchers conducting heart failure omics studies, constituting either seminal works or essential references in this domain. This analytical approach effectively captures: temporal patterns of research attention through burst dynamics, and the core intellectual foundations through high-impact citations, thereby mapping the evolving knowledge structure of heart failure omics research.

By analyzing the co-citation and cited references in HF omics research literature, a co-cited reference diagram (Fig. 10) was generated, encompassing 538 references. Table 6 lists the top 12 most frequently co-cited references. Based on this, a burst detection graph (Fig. 11) was created to identify authoritative references in the field of HF omics research. The most cited reference with the highest burst strength is the “2016 ESC Guidelines for the Diagnosis and Treatment of Acute and Chronic HF,” published in the European Heart Journal by Piotr Ponikowski and the ESC Scientific Document Group in 2016. This underscores the guideline’s authority and reliability in both clinical and foundational research on HF,^[9]^ establishing it as a vital reference for HF researchers globally. In 2023, the Task Force for the Diagnosis and Treatment of Acute and Chronic HF of the European Society of Cardiology once again updated the guidelines,^[4]^ to which we extend our highest respect and admiration for their work.

**Table 6:** Top 12 frequency co-citation representative literature of heart failure omics studies.

Rank	Cited references	Citations	Author	Centrality	Year	Journal
1	2016 ESC Guidelines for the diagnosis and treatment of acute and chronic heart failure: The Task Force for the diagnosis and treatment of acute and chronic heart failure of the European Society of Cardiology (ESC)Developed with the special contribution of the Heart Failure Association (HFA) of the ESC	11	Ponikowski P	0.01	2016	European Heart Journal
2	Epigenome-Wide Association Study Identifies Cardiac Gene Patterning and a Novel Class of Biomarkers for Heart Failure	10	Meder B	0.01	2017	Circulation
3	Cardiovascular Metabolomics	10	McGarrah RW	0.06	2018	Circulation Research
4	Relevance of Multi-Omics Studies in Cardiovascular Diseases	10	Leon-Mimila P	0	2019	Frontiers in Cardiovascular Medicine
5	Effects of spironolactone on serum markers of fibrosis in people at high risk of developing heart failure: rationale, design and baseline characteristics of a proof-of-concept, randomized, precision-medicine, prevention trial. The Heart OMics in AGing (HOMAGE) trial	9	Pellicori P	0	2020	European Journal of Heart Failure
6	Emerging Role of Precision Medicine in Cardiovascular Disease	9	Leopold JA	0.05	2018	Circulation Research
7	Multi-omics approaches to disease	8	Hasin Y	0.15	2017	Genome Biology
8	The effect of spironolactone on cardiovascular function and markers of fibrosis in people at increased risk of developing heart failure: the heart “OMics” in aging (HOMAGE) randomized clinical trial	8	Cleland JGF	0	2021	European Heart Journal
9	Cells of the adult human heart	8	Litvinukova M	0.09	2020	Nature
10	Cardiac Energy Metabolism in Heart Failure	7	Lopaschuk GD	0.06	2021	Circulation Research
11	STRING v11: protein-protein association networks with increased coverage, supporting functional discovery in genome-wide experimental datasets	7	Szklarczyk D	0	2019	Nucleic Acids Research
12	Artificial Intelligence in Cardiology	7	Johnson KW	0.01	2018	Journal of the American College of Cardiology

## 4. Discussion

HF is one of the most intensively researched cardiovascular diseases. In recent years, the prevalence of HF has slightly declined.^[10]^ Numerous guidelines have been dedicated to optimizing the standard medication for HF patients and providing various non-pharmacological treatment options for advanced-stage patients, yet the prognosis remains suboptimal. According to the ASIAN-HF registry, the all-cause mortality rate within one year for symptomatic HF patients is approximately 9.6%.^[11]^ In terms of long-term survival rates, a systematic review involving 1.5 million HF patients revealed that the estimated 5-year and 10-year survival rates post-diagnosis were 56.7% and 34.9%, respectively.^[12]^ Consequently, delving deeper into the mechanisms, identifying novel predictive biomarkers for HF, and achieving personalized treatment for HF have emerged as predominant research directions in the field of HF.

From the perspective of omics research, genetic variations (genomics) can lead to changes in gene expression (transcriptomics), which in turn affect protein variations (proteomics). Based on protein variations, the activity of enzymes within the organism changes, resulting in alterations in metabolites (metabolomics) and metabolic rates (fluxomics).^[13]^ This process is integral throughout the pathological progression of HF. Basic research has found that applying transcriptomics to study the expression of cardiac genes at the RNA level can reveal the pathophysiological processes of cardiovascular diseases.^[14]^ On the other hand, the heart’s beating demands a substantial supply of energy, and most cardiovascular diseases involve cardiac metabolic disturbances. Utilizing metabolomics to capture the dynamic changes of metabolites at the molecular level and exploring the relationship between cardiac pathological alterations and metabolism has emerged as a new research direction.^[15]^ In summary, applying omics technologies to investigate HF aids in unveiling the pathological processes of HF, identifying novel therapeutic targets, and realizing precision medicine.

Based on this, our study utilizes the WoSCC database and applies bibliometric methods alongside visual analysis systems to review the literature on HF omics research over the past 16 years. We aim to uncover the current research status and hotspots in this field, hoping to provide a reference for future studies. The summary is as follows.

### 4.1. Current status of HF omics research

From January 1, 2008, to December 31, 2024, a total of 282 HF omics research articles were collected, published in 169 journals by 622 researchers from 273 institutions across 52 countries. These articles cited 538 references from 454 journals. Over the past 16 years, research interest in this field has consistently increased, with the most significant rise occurring between 2020 and 2021. The continuous release of research findings demonstrates that omics technologies have become one of the essential methods in HF research. Among the countries globally, the United States has the earliest publications and the highest number of publications. Meanwhile, the United Kingdom and China also rank in the top 5 in terms of both publication volume and centrality, indicating that these 3 countries have made substantial contributions and hold significant influence in the field of HF omics research. The leading research institutions in this field include the German Center for Cardiovascular Research, Harvard University, and Brigham and Women’s Hospital. However, as shown in Figures 3 and 4, the centrality among countries and institutions is relatively low, and an international collaboration network has not yet been established. Strengthening cooperation and communication in future research may promote further advancements in this field.

According to the author analysis results, the most prolific author is Zannad, Faiez. His team utilized randomized controlled trials and omics research to discover that spironolactone may influence the metabolism of type I collagen in HF patients. Additionally, they employed proteomics to demonstrate that spironolactone has pleiotropic effects, including reducing cardiac fibrosis, inflammation, thrombosis, congestion, and improving vascular function. These mechanisms may mediate cardiovascular protection and slow the progression of HF.^[16,17]^ His outstanding achievements in HF omics research have provided new insights into the mechanisms by which drugs can improve HF.

Among the authoritative co-cited references, in addition to the aforementioned 2016 European Guidelines for Acute and Chronic HF, another highly influential paper is “Epigenome-Wide Association Study Identifies Cardiac Gene Patterning and a Novel Class of Biomarkers for HF” by Benjamin Meder’s team, published in Circulation in 2017. This study represents the first multi-omics epigenome-wide association research conducted in HF patients. By employing a staged multi-omics approach, it identified epigenetic susceptibility regions and novel biomarkers associated with myocardial dysfunction and HF.^[18]^ This work has had a profound impact on the field of HF omics research.

### 4.2. HF omics research hotspots

Through keyword co-occurrence and clustering analysis, 20 frontier and hotspot keywords in HF omics research were identified (Fig. 8). Among these, “HF” and “cardiovascular diseases” are disease names and thus excluded from discussion; “CHD” and “coronary artery disease” are synonymous, as are “gene expression” and “expression,” so only one term from each pair was included in the analysis. The results indicate that the research hotspots in HF omics can be categorized into 2 main aspects: one elucidates the key research focuses related to omics technologies, and the other highlights cardiovascular diseases closely associated with HF omics research. These findings are summarized and analyzed as follows.

### 4.3. Research technology hotspots

#### 4.3.1. Biomarker

The heat intensity peaks during the years 2018 to 2020, with a burst intensity of 2.85. This keyword first emerged in 2011 and stands as the most intense and earliest appearing keyword in HF omics research, representing a core research hotspot in this field. Biomarkers, discovered in 1989, were initially solely utilized as biological parameters for assessing health status. In research, scholars found that measuring biomarkers could link the impact of interventions on molecules and cells to clinical responses, thereby interpreting clinical study outcomes.^[19]^ Subsequently, biomarkers gradually came to be used as indicators of normal biological processes, pathogenic processes, and pharmacological treatment responses.With the discovery of biomarkers’ specificity and rapidness in acute settings, their primary roles in the diagnosis, prognosis, and selection of specific medications for HF have been established.^[20,21]^ These biomarkers, such as natriuretic peptides (including brain natriuretic peptide (BNP) and NT-proBNP), which are cell stretch biomarkers, have strong clinical applicability and are now recognized and recommended by multiple guidelines. BNP is an important neurohormone secreted by the heart, primarily synthesized and released by ventricular cardiomyocytes in response to volume or pressure overload. Studies have demonstrated that increased ventricular wall tension (such as in HF) significantly stimulates BNP secretion, endowing it with strong clinical applicability. However, the biological system contains numerous heterogeneous biomarkers, making it challenging to identify ideal, specific biomarkers and validate their clinical relevance. Historically, pathological biomarkers for HF were identified through traditional hypothesis-driven approaches.^[22]^ In contrast, omics technologies focus on systematic evaluation of biomolecules, with metabolomics specifically dedicated to comprehensive analysis of low-molecular-weight compounds in organisms, offering potential for novel biomarker discovery.^[23]^ Nicolas Girerd et al employed a nested case-control design across 3 independent cohorts to identify protein biomarkers for incident HF, including the HOMAGE cohort (Heart Omics and Ageing) as the omics research cohort. Their findings demonstrated significant associations across all 3 cohorts for 8 protein biomarkers: BNP, NT-proBNP, 4E-BP1 (4E-binding protein 1), HGF (hepatocyte growth factor), Gal-9 (galectin-9), TGF-alpha (transforming growth factor alpha), THBS2 (thrombospondin-2), and uPAR (urokinase plasminogen activator surface receptor). These biomarkers were ultimately shown to be involved in inflammatory and remodeling pathways.^[24]^ These findings hold important implications for the clinical diagnosis and treatment of HF. As a secondary analysis of HF omics data, this study confirms that the primary objective of HF omics research is to excavate and discover novel biomarkers that can enhance our understanding of disease mechanisms and improve therapeutic strategies. Studies have confirmed that the combined use of standard HF diagnostic biomarkers and metabolomics has significant value in the diagnosis and prognosis of HF.^[25]^

#### 4.3.2. Pathway/receptor

Both of the above 2 keywords have a heat intensity concentrated between 2022 and 2024, with an emergence intensity of 2.48 each. They are the keywords with the highest emergence intensity in HF omics research over the past 3 years, representing current research hotspots. The material and functional causal changes in the pathological process of HF are complex and still contain unknowns. Research on the pathogenesis of most diseases and the mechanisms of drug action primarily focuses on specific pathways and the functions of specific biomolecules, examining changes in proteins, metabolites, ions, and receptors at the cellular level after drug intervention or under pathological conditions. Omics technologies serve as a bridge between mechanistic research and clinical research. Compared to traditional methods that only study literature or single experimental data, omics technologies can investigate the entire process of gene variation and expression, protein and enzyme changes, metabolite and metabolic rate alterations during biological events. They also conduct detailed network analyses of candidate biomarkers based on big data to identify and validate target receptors and pathways, providing evidence for further elucidating the pathophysiological mechanisms of HF and directions for exploring the mechanisms of action of HF-related drugs.^[23]^ A plasma proteomics study by Ferreira JP, including 286 HF patients and 591 healthy individuals, found 38 target proteins that point to 4 pathways: inflammation and apoptosis, extracellular matrix remodeling and angiogenesis, blood pressure and renin-angiotensin system regulation, and atherosclerosis regulation.^[26]^

### 4.4. Cardiovascular diseases with high relevance to HF omics research

#### 4.4.1. Coronary heart disease

The focus on CHD was concentrated between 2011 and 2020, with a burst strength of 2.35, making it the keyword with the longest time span in HF omics research. This demonstrates that CHD is the cardiovascular disease with the highest relevance and the most sustained interest in HF omics studies. CHD, a series of ischemic symptoms caused by coronary atherosclerosis, is the most common cause of ischemic HF. In the United States, approximately 125 million patients with coronary atherosclerosis can be classified as either Stage A HF or at-risk populations for HF.^[1]^ Therefore, CHD can be regarded as an early stage of HF, while HF can be seen as a potential outcome of CHD. Given their close relationship, the 2 are often combined in research. Omics technologies can focus on the progression from CHD to HF, monitoring changes in endogenous proteins and metabolites at different stages or under various interventions. This enables a deeper exploration of mechanisms and the identification of potential biomarkers, paving the way for the development of cardiac protective therapies for acute myocardial ischemia/reperfusion injury and ischemic HF. To analyze the distinct characteristics of HF caused by ischemic etiology versus other causes, Liu et al performed transcriptomic analysis on biological samples from 6 patients (three normal (NF), one with ischemic heart disease (ISCH), and 2 with dilated cardiomyopathy (DCM)). Pairwise comparisons in the test cohort (NF vs ISCH, NF vs DCM, ISCH vs DCM) revealed robust gene expression signatures capable of classifying 313 patients’ failing and non-failing hearts with 90% accuracy, confirming that RNA-seq can differentiate HF caused by different etiologies.^[27]^

#### 4.4.2. Acute myocardial infarction

The focus on AMI was concentrated between 2016 and 2020, with a burst strength of 2.42, making it a keyword with both a significant time span and strong burst intensity in HF omics research. AMI, the most severe manifestation of coronary artery disease, affects over 7 million people globally each year. Although the prevalence of AMI has steadily declined since the 1990s,^[28–30]^ primary ischemic myocardial damage, primarily caused by AMI, remains one of the leading causes of HF.^[31]^ Given the critical role of AMI in the cardiovascular event chain and the changes in myocardial biomarkers resulting from myocardial infarction, the application of omics technologies to study HF in conjunction with AMI has become a major research focus. Henri Charrier et al employed a multi-omics approach, integrating proteomics data from the left ventricles of rats with left coronary artery ligation-induced myocardial infarction and multi-omics data from plasma of post-myocardial infarction patients. Their study predicted that 7 miRNAs, including miR-21-5p, are involved in cardiac development and left ventricular remodeling during the progression from myocardial infarction to HF.^[32]^

#### 4.4.3. Atrial fibrillation

The focus on AF was concentrated between 2018 and 2019, with a burst strength of 2.15. AF is the most common clinical arrhythmia,^[33]^ and HF is the most frequent adverse event triggered by AF.^[34]^ Studies have confirmed that patients with AF complicated by HF have a poorer prognosis, with a mortality risk 3.4 times higher than those without HF.^[35]^ However, the exact mechanisms by which AF induces HF remain unclear, and omics technologies can provide novel research approaches to address this. Haiyu Zhang et al., based on integrated metabolomics and proteomics analysis, identified 10 differential proteins and metabolites, including UBADC1 and quinic acid, as potential biomarkers for AF complicated by HF.^[36]^

## 5. Conclusion

In summary, This study is the first to apply bibliometric methods, utilizing CiteSpace 6.2.R4 software to analyze 282 publications in the field of HF omics from 2008 to 2024. The results indicate that research interest in this field has been increasing year by year, with a corresponding rise in the number of publications. The United States, the United Kingdom, China, Germany, and Italy are the leading countries in this research area. While numerous institutions and researchers are engaged in HF omics studies, the level of collaborative connectivity is relatively weak. Harvard University in the United States stands out as an authoritative research institution, and Zannad, Faiez from the University of Lorraine is a representative researcher in this field. Moving forward, there is potential to enhance collaboration and communication among countries, institutions, and scholars in this domain. Over the past 16 years, research hotspots in HF omics have included AMI, AF, biomarkers, and pathways, demonstrating both depth and breadth in research exploration.

In this study, through comprehensive analysis and discussion of HF omics research, we identify several persistent challenges in this field: First, the cardiovascular system maintains intricate connections with multiple vital organs. As the terminal stage of cardiovascular diseases, HF patients frequently present with comorbidities involving other organ systems, particularly pulmonary diseases. The heart and lungs exhibit particularly strong pathophysiological interdependence. Smoking, for instance, directly damages pulmonary tissue while simultaneously serving as an independent risk factor for coronary artery disease. Patients with chronic obstructive pulmonary disease (COPD) typically demonstrate moderate to severe chronic airflow limitation and exhibit higher baseline incidence of cardiac pathologies, with disease progression mechanisms remarkably similar to those observed in HF.^[37–40]^ A retrospective analysis of 3730 severe COPD patients employing a nomogram model confirmed significant COPD-HF comorbidity, identifying advanced age as a shared risk factor while highlighting the need for cautious clinical application of diuretics and mechanical ventilation in COPD-HF patients.^[41]^ These findings underscore the profound clinical interconnection between COPD and HF. Notably, our keyword burst analysis failed to identify research hotspots involving other organ systems. We attribute this to our study’s exclusive focus on “HF” during literature screening, which systematically excluded investigations targeting other systemic diseases. Nevertheless, we contend that future HF omics research should transcend isolated HF investigations by prioritizing HF comorbidities and multi-organ end-stage pathophysiology to better reflect clinical realities and improve patient management. Second, omics research involves extensive biomarker detection and analysis of clinical biospecimens, generating massive datasets that pose substantial ethical challenges. Critical unresolved issues include: whether researchers have obtained formal approval from medical ethics committees regarding study design and methodology; whether operational protocols strictly adhere to ethical requirements, including proper informed consent acquisition and rigorous patient privacy protection. We strongly advocate for strict compliance with ethical guidelines to ensure responsible research conduct.

This study has the following limitations: First, due to the constraints of the CiteSpace software, the bibliometric analysis only included relevant literature from the WoSCC, excluding publications from other databases. Additionally, factors such as publication time and language may result in incomplete literature coverage and potential biases in the findings. Second, regarding the current state of research in the field of HF omics, the visual analysis of literature serves as a preliminary exploration and requires further research and analytical validation. Moreover, compared to other types of research, the current volume of studies in HF omics remains relatively limited. The study anticipates that future research could incorporate a larger literature base to further explore hotspots and development trends in this field, thereby promoting disciplinary advancement.

## Acknowledgments

The authors would like to thank all the reviewers who participated in the review.

## Author contributions

**Conceptualization:** Lin zhou, Jiankang Wang, Yongsheng Huang, Jiajuan Guo.

**Data curation:** Jiankang Wang, Yongsheng Huang.

**Formal analysis:** Hang Shang, Jiayu Mao.

**Investigation:** Hang Shang, Wenxuan Wang.

**Methodology:** Wenxuan Wang.

**Writing – original draft:** Lin zhou, Zihan Wang.

**Writing – review & editing:** Lin zhou, Zihan Wang, Yingzi Cui.
